# Identification of cellular proteome using two-dimensional difference gel electrophoresis in ST cells infected with transmissible gastroenteritis coronavirus

**DOI:** 10.1186/1477-5956-11-31

**Published:** 2013-07-16

**Authors:** Xin Zhang, Hong-Yan Shi, Jian-Fei Chen, Da Shi, Hong-Wu Lang, Zhong-Tian Wang, Li Feng

**Affiliations:** 1 Division of Swine Infectious Diseases, State Key Laboratory of Veterinary Biotechnology, Harbin Veterinary Research Institute, Chinese Academy of Agricultural Sciences, No. 427 Maduan Street, Nangang District, Harbin 150001, China; 2 Chinese Institute of Veterinary Drug Control, Beijing 100081, China

**Keywords:** Proteome, ST cell, DIGE, TGEV

## Abstract

**Background:**

Transmissible gastroenteritis coronavirus (TGEV) is an enteropathogenic coronavirus that causes diarrhea in pigs, which is correlated with high morbidity and mortality in suckling piglets. Information remains limited about the comparative protein expression of host cells in response to TGEV infection. In this study, cellular protein response to TGEV infection in swine testes (ST) cells was analyzed, using the proteomic method of two-dimensional difference gel electrophoresis (2D DIGE) coupled with MALDI-TOF-TOF/MS identification.

**Results:**

33 differentially expressed protein spots, of which 23 were up-regulated and 10 were down-regulated were identified. All the protein spots were successfully identified. The identified proteins were involved in the regulation of essential processes such as cellular structure and integrity, RNA processing, protein biosynthesis and modification, vesicle transport, signal transduction, and the mitochondrial pathway. Western blot analysis was used to validate the changes of alpha tubulin, keratin 19, and prohibitin during TGEV infection.

**Conclusions:**

To our knowledge, we have performed the first analysis of the proteomic changes in host cell during TGEV infection. 17 altered cellular proteins that differentially expressed in TGEV infection were identified. The present study provides protein-related information that should be useful for understanding the host cell response to TGEV infection and the underlying mechanism of TGEV replication and pathogenicity.

## Background

Transmissible gastroenteritis virus (TGEV) is a member of the *Coronaviridae* family [1]. The infection of TGEV causes severe diarrhea in suckling piglets (about 2 weeks old) and its lethality approaches 100%, which results in enormous economic loss in swine-producing areas in the world [2]. TGEV is an enveloped virus with a positive-sense RNA genome of 28.5 kb. About two-thirds of the TGEV genome encodes the replicase proteins (rep) at the 5′ end, and one-third of the genome encodes other viral genes at the 3′ end in an order of 5′-S-3a-3b-E-M-N-7-3′ [3]. The genome of TGEV encodes four structural proteins: spike (S), membrane (M,), minor envelope (E), and nucleocapsid (N) proteins. The surface protein S, a large type I transmembrane glycoprotein that forms peplomers, is responsible for cell receptor binding and membrane fusion [4]. The M protein spans the membrane and interacts with the N protein to form core of the virus during assembly [5,6]. The small E protein, a transmembrane protein detected as a minor structural component, is essential for TGEV replication [7]. The N protein, an internal phosphoprotein [8], interacts with the TGEV genomic RNA to form viral nucleocapsid [5,9], and may disrupt host cell division [10]. To date, there is limited information about host cell responses to TGEV infection.

Proteomics analysis enables a more comprehensive characterization of virus-virus and virus-host interactions involved in infection and pathogenesis [11,12]. The development of proteomic approaches have greatly facilitated detection of proteins induced in virus infected cells. Among those techniques for differentially expressed protein spot analysis, two-dimensional difference gel electrophoresis (2D DIGE) is reproducible and sensitive [13]. 2D DIGE has greatly facilitated the comparison of two samples by removing gel-to-gel variability and by using dyes (CyDye) with a greater dynamic range than traditionally used silver or Coomassie stains [14]. Using 2D DIGE method followed by mass spectrometry (MS) identification, comparative proteomics of host cells have been investigated during virus infection, including human influenza A virus [15], human immunodeficiency virus type 1 (HIV-1) [16], hepatitis B virus [17], hepatitis C virus (HCV) [18], Epstein-Barr virus (EBV) [19], and Dengue virus (DENV) [20], and porcine reproductive and respiratory syndrome (PRRSV) [21]. Proteomics method analyzing host cellular responses to TGEV infection can be used to identify important cellular factors involved in viral pathogenesis.

In present study, to determine protein profiles of swine testes (ST) cell line that expressed differentially after TGEV infection, fluorescent 2D DIGE coupled with a MALDI-TOF-TOF/MS identification proteomics approach was utilized. A total of 33 differentially expressed protein spots were identified, and those proteins of interest were confirmed by Western blot. This study can provide useful clues for better understanding of TGEV replication and pathogenesis.

## Results

### Confirmation of TGEV propagation in ST cells

**Figure 1:**
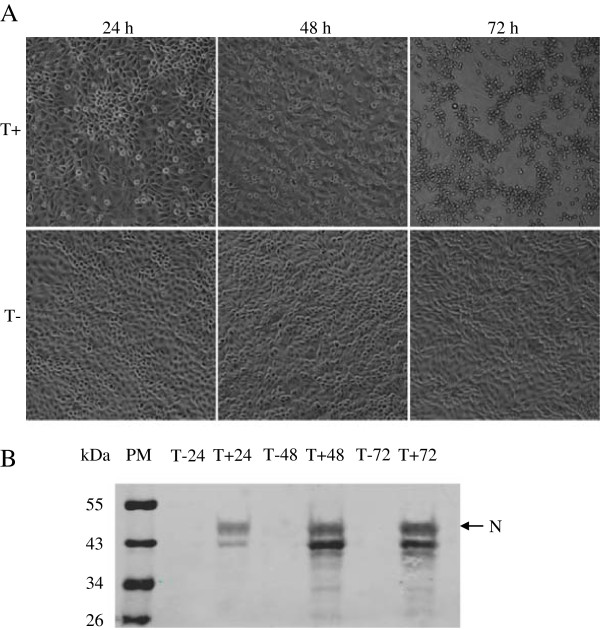
**Cytopathic effect caused by TGEV infection and identification of the TGEV infected and non-infected ST cells.** The cytophatic effect induced by the TGEV virus was analyzed by optical microscopy, at 24 h, 48 h and 72 h p.i. Images were taken with a 40× objective (**A**). The N protein in TGEV-infected and mock-infected ST cells were checked using the mAb to N protein of the TGEV by the method of Western blot (**B**). T + and T- represent the TGEV infected and uninfected ST cells, respectively.

To obtain a detailed comparison of differences in protein expression, the cellular proteins were extracted at 24 h, 48 h and 72 h p.i. from the TGEV-infected and mock-infected ST cells and were identified by IFA and Western blot analysis using mAb to N protein of TGEV as primary antibody. IFA and Western blot analysis revealed that the ST cells infected with TGEV could be recognized with mAb to N protein of the TGEV at 24 h, 48 h and 72 h p.i. (Figure 1). To determine the host response of ST cells following TGEV infection, the cytophatic effect (CPE) was also observed at 24 h, 48 h, and 72 h p.i. TGEV induced significant CPE at 48 h and 72 h p.i., characterized by rounding and detachment of the cells (Figure 1A). TGEV-infected ST cells at 48 h p.i. were chosen for subsequent proteomics analysis, in which a high percentage of TGEV-infected cells showed considerable cell death.

### Analysis of differentially expressed proteins after TGEV infection

**Figure 2:**
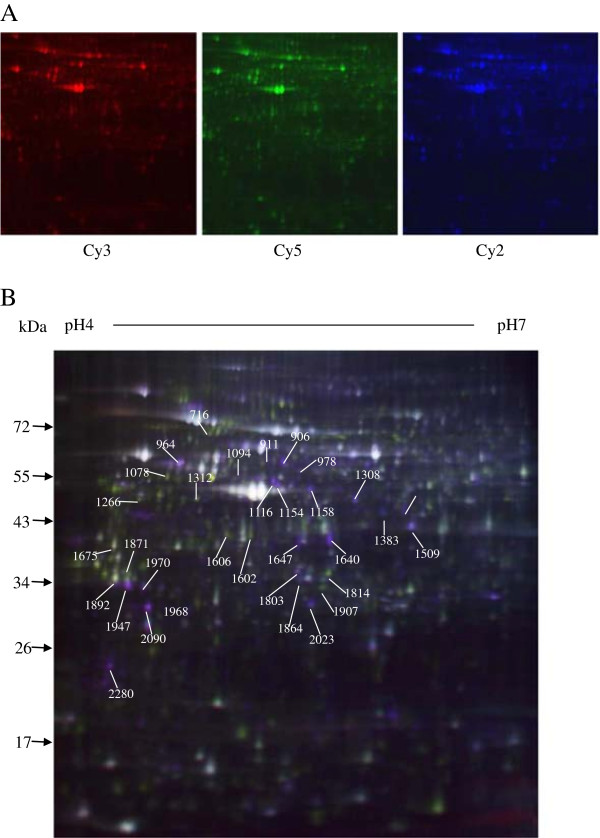
**Differentially expressed protein spots (marker with master numbers) displayed in 2D DIGE images.** The TGEV-infected ST cell lysates are labeled with Cy3 (red), the mock-infected ST cells are labeled with Cy5 (blue), the internal standard proteins are labeled with Cy2 (yellow) **(A)**. Representative data from a 2D DIGE **(B)**.

**Figure 3:**
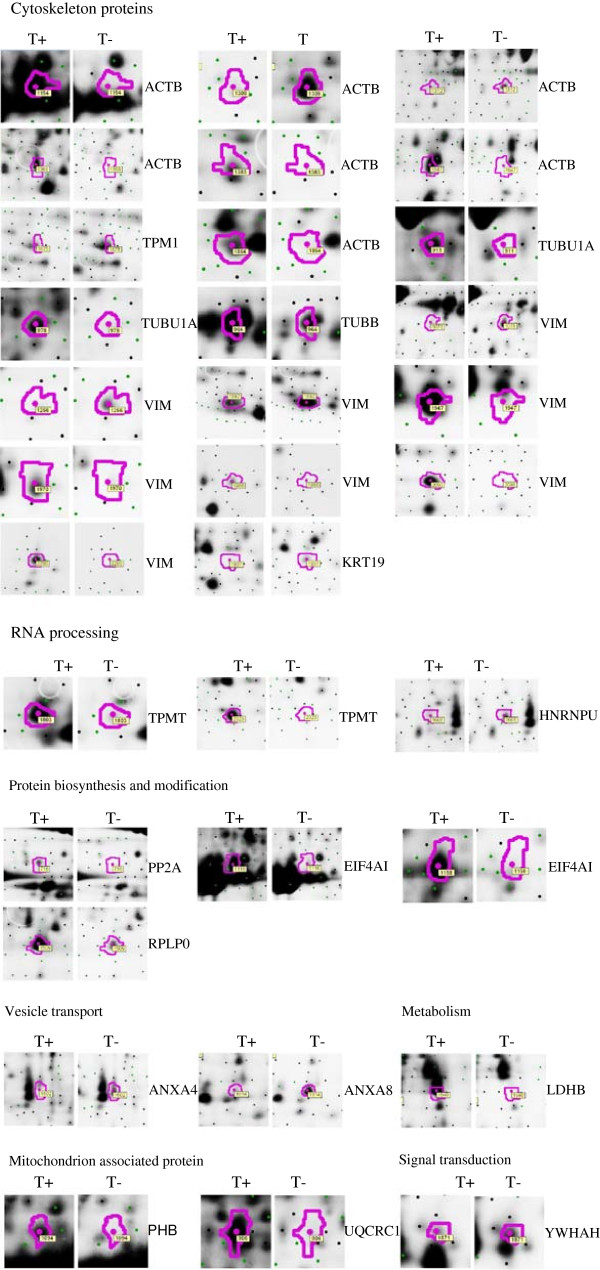
**Image of ImageQuant and DeCyder analysis of 35 differentially expressed protein spots in 2D DIGE gels.** T + and T- represent TGEV-infected and mock-infected ST cells, respectively.

To identify cell proteins involved in response to TGEV infection, 2D DIGE proteomics method was performed. Three independent experimental repeat of TGEV-infected and mock-infected ST cells were included in this analysis (Additional file 1: Figure S1). After 2D DIGE, the Cy2, Cy3, and Cy5 channels of each gel were individually imaged, and were analyzed using Decyder software package (version 6.04.11, GE Healthcare). For proteins separated in the pH 4–7 range, 2,710 protein spots were matched. Of which, 33 spots were significantly modified between the TGEV-infected and mock-infected ST cells (2-fold difference in abundance and p < 0.05), including 23 spots that were up-regulated and 10 that were down-regulated (Figure 2 and Figure 3).

### Mass spectral identification of differentially expressed proteins

**Figure 4:**
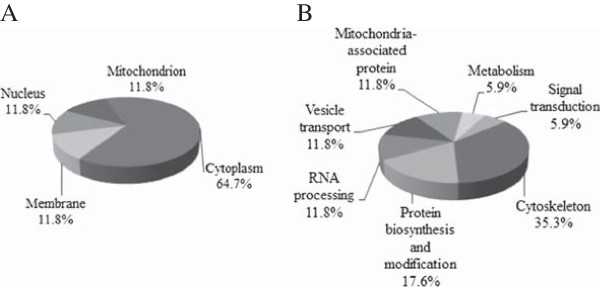
**Pie chart representing differentially expressed proteins identified by mass spectrometry following TGEV infected ST cells.** Proteins were classified according to their subcellular location (**A**) and biological function (**B**).

To identify the differentially expressed proteins in TGEV-infected ST cells, a total of 33 protein spots with a threshold greater than 2-fold were excised manually from gels and subjected to in-gel trypsin digestion and subsequent MALDI-TOF/TOF identification. As shown in Figure 4 and Table 1, 33 differentially expressed protein spots (Additional file 2: Table S1), comprising 23 up-regulated and 10 down-regulated protein spots, were successfully identified (the MS and MS/MS spectra are listed in Additional file 1: Figure S1). According to the protein function and subcellular annotations from the Swiss-Prot and TrEMBL protein database and Gene Ontology Database, the identified cellular proteins were comprised of cellular structure and integrity, RNA processing, protein biosynthesis and modification, vesicle transport, signal transduction, and mitochondrial pathway.

**Table 1:** Proteins identified from the differential 2D DIGE analysis after TGEV infection

**Spot number**^**a**^	**Protein name (Abbr.)**	**Accession number **^**b**^	**Molecular mass** **(kDa)**	**pI**	**Peptide matched **^**c**^	**Sequence coverage (%)**	**Protein score **^**d**^	**(TGEV/mock)**	
								**Average volume ratio **^**e**^	***p *value **^**e**^
Cytoskeleton proteins									
Microfilament-associated proteins									
1154	beta actin (ACTB)	gi|45269029	44.76	5.55	3	36	285	3.64	0.000035
1308	beta actin (ACTB)	gi|45269029	44.76	5.55	3	16	407	4.04	0.00013
1312	beta actin (ACTB)	gi|45269029	44.76	5.55	3	14	129	−2.82	0.00076
1368	beta actin (ACTB)	gi|45269029	44.76	5.55	1	16	82	4.68	0.0097
1383	beta actin (ACTB)	gi|45269029	44.76	5.55	5	34	537	3.7	0.0019
1647	beta actin (ACTB)	gi|150438831	44.76	5.55	3	21	242	7.03	0.005
1675	alpha tropomyosin (TPM1)	gi|158931149	32.69	4.71	5	46	544	−2.82	0.0027
1864	beta actin (ACTB)	gi|476332	26.10	5.55	3	35	177	2.58	0.00023
Microtubule-associated proteins									
911	alpha Tubulin (TUBA1B)	gi|116256086	50.12	4.94	4	28	340	9.08	0.0029
964	beta Tubulin (TUBB)	gi|75045190	49.64	4.78	7	31	448	2.28	0.00081
978	alpha Tubulin (TUBA1B)	gi|116256086	50.12	4.94	3	32	338	3.38	0.000014
Intermediate filament proteins									
1078	vimentin (VIM)	gi|335296459	53.64	5.06	6	64	1020	−3.26	0.0001
1266	vimentin (VIM)	gi|335296459	53.64	5.06	6	60	888	−4.28	0.00013
1892	vimentin (VIM)	gi|335296459	53.64	5.06	2	35	389	−2.82	0.00041
1947	vimentin (VIM)	gi|335296459	53.64	5.05	4	35	582	4.84	0.000014
1968	vimentin (VIM)	gi|21431723	30.97	4.67	4	25	366	3.06	0.00036
1970	vimentin (VIM)	gi|335296459	53.64	5.06	4	35	603	2.43	0.00082
1907	keratin 19 (KRT19)	gi|311267276	44.19	5.05	8	47	682	−3.99	0.000023
2090	vimentin (VIM)	gi|21431723	30.97	4.67	2	9	120	5.63	0.0095
2280	vimentin (VIM)	gi|335296459	53.64	5.06	6	34	504	9.65	0.0031
RNA processing									
1606	heterogeneous nuclear ribonucleoprotein U (HNRNPU)	gi|335296158	104.85	5.85	3	7	277	−3.26	0.011
1803	thiopurine S-methyltransferase (TPMT)	gi|311259781	28.46	5.46	5	41	497	3.59	0.000099
2023	thiopurine S-methyltransferase (TPMT)	gi|311259781	28.46	5.46	5	54	476	6.96	0.000008
Protein biosynthesis and modification									
716	protein phosphatase 2A (PP2A)	gi|510469	65.28	5.00	2	30	239	2.71	0.0051
1116	eukaryotic initiation factor 4A-I (EIF4A1)	gi|154147660	46.13	5.32	7	44	586	3.87	0.00052
1158	eukaryotic initiation factor 4A-I (EIF4A1)	gi|154147660	46.13	5.32	5	40	531	3.37	0.0011
1509	acidic ribosomal protein P0 (RPLP0)	gi|182705234	34.34	5.71	4	50	483	2.38	0.02
Vesicle transport									
1602	annexin A8 (ANXA8)	gi|194042330	36.71	5.20	6	63	752	−3.31	0.0063
1814	annexin A4 (ANXA4)	gi|4033507	35.81	5.71	5	49	510	−5.96	0.000008
Mitochondrion associated protein									
906	cytochrome b-c1 complex subunit 1 (UQCRC1)	gi|335299041	52.67	5.76	4	38	342	7.9	0.000023
1094	prohibitin (PHB)	gi|335308255	28.88	5.74	6	72	688	3.04	0.0031
Signal transduction									
1871	14-3-3 protein eta (YWHAH)	gi|194043292	28.19	4.81	4	30	282	−2.52	0.00035
Metabolism									
1640	L-lactate dehydrogenase B chain (LDHB)	gi|164518958	36.42	6.73	2	22	126	5.74	0.0016

^a^ Spot number correspond to the unique sample spot number as indicated Figure 2.

^b^ Accession numbers according to NCBInr database.

^c^ Number of peptides identified by MALDI-TOF/TOF is given by MASCOT.

^d^ Protein scores greater than 59 were considered successfully identified (p < 0.05).

^e^ Paired average volume ratio and p values (t test) between ST cells infected by TGEV versus uninfected cells (TGEV/mock) were quantified using DeCyder software.

Thirty-three differential protein spots corresponded to 17 proteins, including cytoskeleton-associated proteins (35.3%), protein biosynthesis and modification proteins (17.6%), RNA processing proteins (11.8%), vesicle transport proteins (11.8%), mitochondria-associated proteins (5.9%), signal transduction-associated proteins (5.9%), and 5.9% metabolism-associated proteins. These proteins (Figure 3) were mainly located in the cytosol (64.7%), membrane (11.8%), nucleus (11.8%), and mitochondrion (11.8%). In addition, some different spots were identified to be products of the same gene, including beta actin, alpha tubulin, vimentin, eukaryotic initiation factor 4A-I, and thiopurine S-methyltransferase. In particular, 7 protein spots (6 up-regulated spots and 1 down-regulated spot) were identified as beta actin, and 8 protein spots (5 up-regulated spots and 3 down-regulated spots) were identified as vimentin.

### Analysis of identified proteins at the transcriptional level

**Figure 5:**
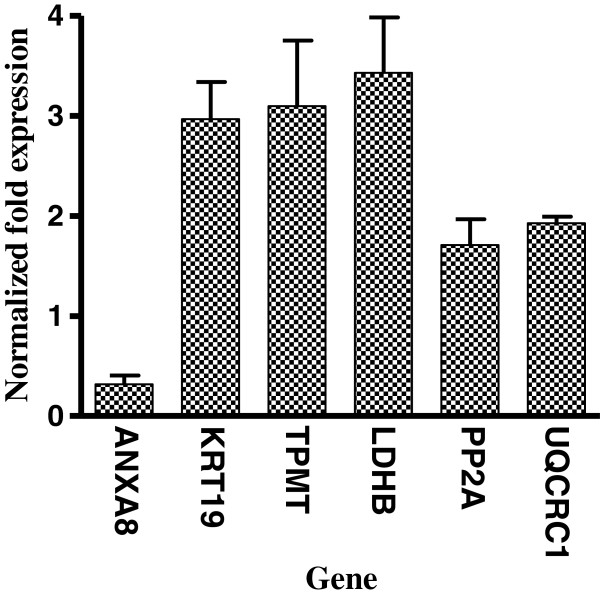
**Transcript alteration of the differentially expressed proteins in TGEV-infected ST cells.** Total cellular RNA of ST cells with or without TGEV infection at 48 h p.i. was measured by real time RT-PCR analysis. Samples were normalized with GAPDH gene as a control housekeeping gene. Error bars represent standard deviation. Gene symbols indicating different genes refer to Table 1 or Table 2.

Alterations in expression of a protein may be due to a change in its mRNA level. In order to confirm the results of the proteomics analysis at the mRNA level, the transcriptional alterations in six selected proteins were measured by real-time RT-PCR. Glyceraldehyde-3-phosphate dehydrogenase (GAPDH) gene was used as a control housekeeping gene. The mRNA level of ANXA8 was decreased in TGEV-infected ST cells (Figure 5). The mRNA level of KRT19, TPMT, LDHB, PP2A, UQCRC1 were increased in TGEV-infected ST cells (Figure 5). The trends of change in the mRNA level of ANXA8, TPMT, LDHB, PP2A, and UQCRC1 were consistent with 2D DIGE results. Interestingly, KRT19 had contrary results to with those of 2D DIGE methods. These data provide transcriptional information complementary to those differentially-expressed proteins detected by 2D DIGE analysis.

### Protein validation by western blot

**Figure 6:**
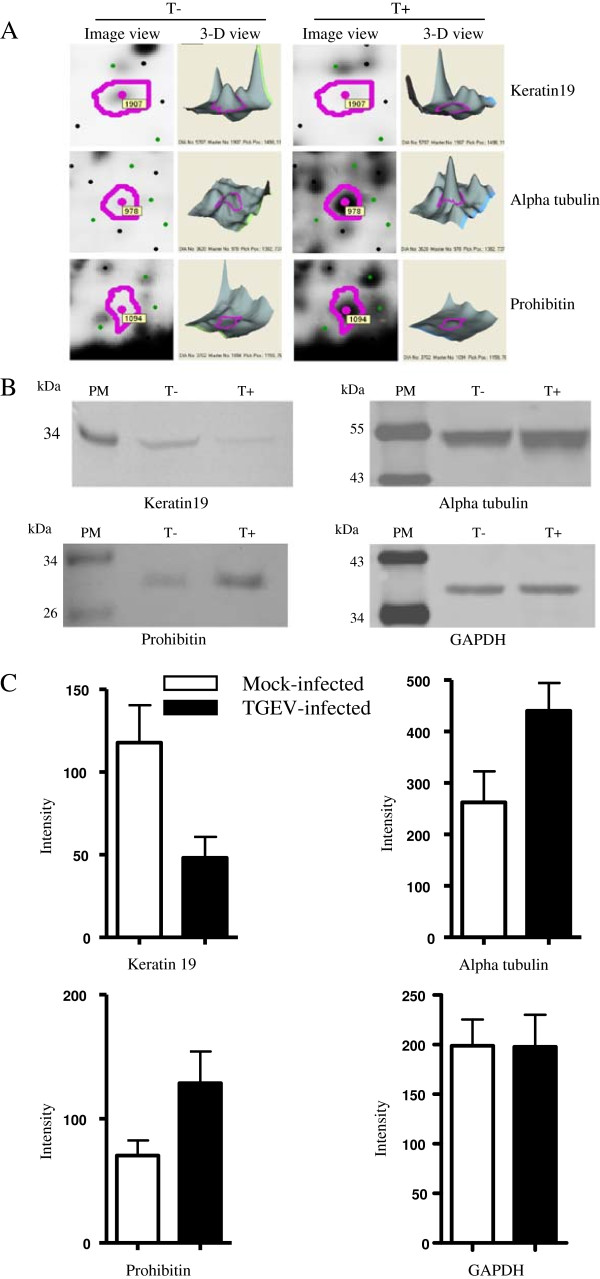
**Western blot confirmation of representative proteins in TGEV-infected ST cells.** Representative image of ImageQuant and DeCyder analysis of keratin19, alpha tubulin, vimentin, and prohibitin in 2D DIGE gels (**A**). The immunoblot analysis of keratin19, alpha tubulin, vimentin, and prohibitin in TGEV-infected and mock-infected ST cells (**B**). The averaged densitometric intensity of keratin19, alpha tubulin, vimentin, and prohibitin in immunoblot analysis, with GAPDH as a loading control (**C**). T + and T- represent TGEV-infected and mock-infected ST cells, respectively. PM, protein marker. Image view and 3-D view obtained from DeCyder.

To further verify those proteins identified by 2D DIGE and MALDI-TOF/TOF mass spectrometry, Western blot analyses were performed. Three proteins, alpha tubulin, keratin 19, and prohibitin were selected for western blot analysis. Equal amounts of cell lysates from TGEV-infected and mock-infected ST cells at 48 h p.i. were examined with specific antibodies to alpha tubulin, keratin 19, and prohibitin. The data showed in Figure 6 indicate that alpha tubulin, keratin 19, and prohibitin were recognized with respective antibodies. From Figure 6, we can see that keratin 19 was down-regulated and alpha tubulin and prohibitin were up-regulated, which was consistent with the 2D DIGE analysis. These data validated the MALDI-TOF/TOF identification of those proteins in TGEV-infected ST cells that differentially expressed.

## Discussion

Proteomics is a novel methodology to detect components of cellular protein interactions as well as host cellular pathophysiological processes that occur during virus infection [11]. Until present investigation, no results have been reported for performing analysis of differential proteome of host cells infected with TGEV. In this study, 2D DIGE coupled with MALDI-TOF/TOF was used to analyze the differential proteome of ST cells infected with TGEV. The 33 differential protein spots were successfully identified as 17 proteins, of which function in diverse biological processes.

Cytoskeletal protein expression was altered in TGEV-infected ST cells. The cytoskeleton filaments are dynamic and divided into three types: microfilaments (actin filament), microtubules, and intermediate filaments [22]. The intermediate filaments can provide mechanical stability to cells, while actin and microtubule cytoskeletons are responsible for trafficking of numerous endogenous cargos as well as intracellular microorganisms throughout the cells [23]. Many viruses use the cytoskeleton for infection and replication, such as HIV-1 [24]. In present study, differentially expressed microfilament-associated proteins beta actin and alpha tropomyosin, microtubule-associated alpha tubulin and beta tubulin, as well as the intermediate filament-associated vimentin and keratin 19 were identified (Table 1), indicating that TGEV infection and replication involves cellular skeleton.

The actin and microtubule cytoskeleton play important roles in the life cycle of viruses [25]. Numerous viral proteins interact with actin-binding proteins or directly with actin [26]. Microtubules and microtubule-associated proteins are known to play important roles in intracellular trafficking of viral components as well as virions in the infected host cell [23]. In this study, the up-regulated microfilament-associated proteins beta actin and microtubule-associated alpha tubulin and beta tubulin were identified, which was believed to facilitate the transport of viral proteins of TGEV from rough endoplasmic reticulum (ER) and Golgi apparatus to the reservoir for viral replication.

Vimentin is a major component of type III intermediate filaments found in many cell lines [27]. This protein serves to maintain cell shape and is involved in attachment, migration and cell signaling [28]. Major changes in the distribution of vimentin are observed when the cell moves and divides [29], but this protein is also redistributed in cells expressing misfolded proteins and during virus infection [30]. Previous studies have shown that intermediate filament protein vimentin was cleaved by human immunodeficiency virus type 1 protease (HIV-1 PR) [31] and that vimentin networks collapsed and was dispersed in IBDV-infected cells [32]. In present study, 8 differential protein spots were identified as vimentin including 5 up-regulated protein spots and 3 down-regulated protein spots. Further study is required to determine whether TGEV papain-like protease 1 (PL1^pro^) [33] cleaves vimentin into different isoform or subunit, using a similar strategy as HIV.

Among those differentially expressed host proteins, some are known to participate in viral replication and translation (Table 1). Positive-strand RNA viruses must recruit normal components of host cellular RNA processing or translation machineries for viral RNA synthesis and protein synthesis [34]. Heterogeneous nuclear ribonucleoprotein U (HNRNPU) is an abundant, strictly nuclear phosphoprotein that interacts directly with RNA through a carboxy-terminal RGG sequence [35]. HNRNPU is known to influence pre-mRNA processing, mRNA transportation to cytoplasm, intracellular localization, translation, and turnover of mRNAs [36]. Previous studies have shown that the levels of HIV-1 viral transcripts are dramatically down-regulated in cytoplasm of infected cells by HNRNPU [37]. In this study, the down-regulated HHRNPU was found after TGEV infection, which may facilitate the replication of viruses. Another RNA processing protein, thiopurine S-methyltransferase (TPMT), was found to be more abundant in TGEV-infected cells. TPMT is a cytoplasmic transmethylase present in prokaryotes and eukaryotes, which has a molecular mass of 28 kDa and comprises 245 amino acids [38]. TPMT is a drug-metabolizing enzyme widely expressed in mammalian and non-mammalian cells [39]. Previous works have reported that TPMT plays a role in BVDV virus replication and thiopurines inhibit bovine viral diarrhea virus production in a TPMT-dependent manner [40]. Based on these data, an up-regulation of TPMT in TGEV-infected ST cells suggests that this host protein plays an important role in TGEV biology, making it possible target for future drug development.

Viruses rely on the cellular translation machinery to translate their own proteins, which facilitates the rapid production of viral proteins and renders an inhibitory effect on the production of host proteins, including host anti-viral proteins [41]. Translation factors have been well documented as playing crucial roles in viral RNA and protein synthesis [42]. In herpes simplex virus type 1 (HSV-1) infected HeLa cells, the synthesis of several ribosomal proteins and their assembly into ribosomes continue in spite of a general inhibition of cellular protein synthesis [43]. Acidic ribosomal protein P0 (RPLP0) is located in the active part of the ribosome particle, at which mRNAs, tRNAs and translation factors interact during protein synthesis [44]. The cellular RPLP0 was observed as up-regulated after virus infection, such as pseudorabies virus (PrV) infected bovine kidney cells [45], and Epstein-Barr virus (EBV) infected primary B cells [46]. In this study, the up-regulated 60S RPLP0 was also found after TGEV infection, suggesting the ribosomal protein plays an important role in the translation of TGEV viral proteins.

Viruses may inhibit host protein synthesis by targeting multiple steps in the gene expression process via various pathways, for instance, the vesicular stomatitis virus (VSV) M protein inhibits the initiation of the transcription of host genes [47] and the SARS-CoV spike protein inhibits host cell translation by interaction with eIF3f [48]. In this study, the up-regulated eukaryotic initiation factor 4A-I (EIF4A1) was identified after TGEV infection. The translation initiation proteins observed in TGEV infected ST cells may be a reflection of translation regulation mechanisms exploited by TGEV virus, interfering with cellular protein synthesis and translation initiation of the host cell for beneficial reasons, which need to be further studied.

Protein phosphatase 2A (PP2A) is an evolutionarily conserved enzyme that represents a major portion of serine/threonine phosphatase activity in cell extracts [49]. PP2A enzymes have been clearly involved in regulation of cell transcription, cell cycle and viral transformation [50]. Up-regulation of PP2A scaffold subunit A and subsequent dephosphorylation of Tyr-307 in the catalytic subunit was found, suggesting PP2A activation in Huh7 infected cells [51,52]. Activation of serine-threonine PP2A was found in Huh7 cells upon HSV-1 infection, and PP2A activation paralleled dephosphorylation and inactivation of the downstream mitogen-activated protein (MAP) kinase pathway [53]. In this study, the up-regulation of PP2A was found in TGEV infected ST cells, suggesting PP2A plays an important role in the dephosphorylation of cellular and viral protein during TGEV infection.

Several proteomics analysis about coronavirus had been done including SARS-CoV [54], IBV [55,56], and MHV [57]. By comparing the finding proteins in this study to previous findings, there is no common gene of target related to coronavirus. The findings in TGEV infected ST cell might not reflect the interaction between the virus and pig intestine epithelial cell. It is surprising that the proteome responses observed did not reveal any immune responses related proteins in TGEV-infected ST cells. It may be related to the host cells chosen or the fact that attenuated TGEV strain was used. Identification of cellular proteome in pig intestine epithelial cell or immune cells infected with TGEV need to be further studied.

## Conclusions

In conclusion, a total of 17 altered cellular proteins that differentially expressed in TGEV infection were identified in this study. Most of these proteins were involved in transcription and translation processes, vesicle transport, signal transduction, and alteration of the cytoskeleton networks. Western blot analysis of alpha tubulin, keratin 19, and prohibitin validated the MALDI-TOF/TOF identification of the differentially expressed proteins in the TGEV-infected ST cells. The present study provides large scale protein-related information that should be useful for understanding the pathogenesis of TGEV infection.

## Materials and methods

### Cell culture, virus infection and sample preparation

The TGEV strain attenuated H (Accession NO. EU074218) [58] was propagated on a ST cell monolayer. The proteins of TGEV infected ST cells were extracted according to the methods previously described [59]. Briefly, the ST cells were infected with attenuated H (H167) at a multiplication of infection (MOI) of 1, and the cells were scraped using a cell scraper at 48 h postinfection (p.i.), and centrifuged at 10,000 × g for 5 min. After washing three times with ice-cold phosphate-buffered saline (PBS), the collected cells were lysed with lysis buffer (7 M urea, 2 M thiourea, 4% [w/v] CHAPS, 65 mM DTT, 0.2% pharmalyte 4/7 and 1 mM PMSF) containing 1% nuclease mix in the final concentration and were vertically vibrated until the cells were completely lysed. The supernatant was collected after centrifuging at 12,000 × g at 4°C for 60 min. Samples were treated with a 2D clean-up kit (GE Health Care) and a 2D quant kit (GE Health Care) according to the instructions of the manufacturers. Paralleled mock-infected ST cells were used as control. Three biological **replicates** of TGEV-infected and mock-infected ST cells were prepared.

### 2D DIGE

A total of 200 pmol of CyDye DIGE Flours (GE healthcare, Germany) were used to label 50 μg of protein samples. To access biological variation, three experimental duplicates were carried out using the samples prepared above. The internal standards (equal amounts of both samples) were labeled with Cy2. Protein extracts from mock-infected ST cells, used as a reference state, were labeled with Cy5. Protein extracts from TGEV-infected ST cells were labeled with Cy3, representing the test states. IEF was performed using an IPGphor system (GE Healthcare) and commercially available 24-cm long IPG strips (Linear, pH 4–7, GE Healthcare). The settings and conditions for active rehydration of the IPG strips were used as previously described [60]. Briefly, IEF was performed using the following parameters: 30 V, 12 h; 200 V, 1 h; 1,000 V, 1 h; 8,000 V, 2 h; and 8,000 V, 65,000 vh. The isoelectric-focused proteins in strips were incubated for 15 min in the equilibration buffer (6 M urea, 30% glycerol, 2% SDS, and 0.375 M Tris, pH 8.8) containing 1% DTT, followed by additional equilibration for 15 min in the equilibration buffer containing 2.5% iodoacetamide. The second dimension separation was performed using the Ettan Dalt II system (GE healthcare). Gels were poured between low fluorescent glass plates, of which one plate was bind-silane treated. Three parallel gels were run at 12°C (running buffer: 25 mM Tris, 192 mM glycine and 1% SDS). The equilibrated IPG strips were further resolved with 12% SDS-PAGE gels at 1 W/gel for 30 min and then 6 W/gel until the dye front reached the bottom of the gels.

### Image acquisition and analysis of 2D DIGE gels

Cy2-labeled, Cy3-labeled, and Cy5-labeled protein images were scanned directly between the low fluorescent glass plates using a Typhoon Variable Mode Imager 9400 (GE Healthcare) with the CyDye-specific settings for excitation at 488 nm, 532 nm and 633 nm, and for emission at 520 nm, 590 nm and 680 nm. All gels were scanned with a resolution of 100 μm and a standard pixel volume of 60,000-80,000 for all scans. Determination of protein abundance and statistical analysis was performed using the Decyder™ software package (version 6.04.11, GE Healthcare). Inter-gel matching performed using the Biological Variation Analysis (BVA) mode. Matching between gels was performed using the in-gel standard from each image pair. A paired *t* test was used for the methods of statistical analysis. Only protein spots showing significance (p < 0.05) and at least a 2-fold difference in abundance (ratio of the mean of the normalized spot volume of the TGEV-infected samples versus mock-infected samples) were considered as up-regulated (ratio > 2) or down-regulated (ratio < −2).

### Protein identification by MALDI-TOF-TOF mass spectrometry (MS) and a database search

For identification of protein spots a preparative gel containing 800 μg of protein was run as described above and stained with PhastGel™ Blue R (GE Healthcare). The protein spots of interest were manually excised from the gels and plated into 96-well microplates. Excised spots were firstly destained twice with 60 μl of 50 mM NH4HCO3 and 50% acetonitrile (ACN) and then dried twice with 60 μl of ACN. Afterwards, the dried pieces of gels were incubated in ice-cold digestion solution (trypsin 12.5 ng/μl and 20 mM NH4HCO3) for 20 min and then transferred into a 37°C incubator for digestion overnight. Finally, peptides in the supernatant were collected after extraction twice with 60 μl extract solution (0.1% TFA in 50% ACN). The peptide solution was dried under the protection of N2, and 0.8 μl matrix solution (5 mg/ml α-cyano-4-hydroxy-cinnamic acid diluted in 0.1%TFA, 50%ACN) was pipetted to dissolve it. Then the mixture was spotted on a MALDI target plate (Applied Biosystems). MS analysis of peptides was performed on an AB SCIEX 5800 MALDI-TOF/TOF. The UV laser was operated at a 400 Hz repetition rate with wavelength of 355 nm. The accelerated voltage was operated at 20 kV and mass resolution was maximized at 1,600 Da. Myoglobin digested with trypsin was used to calibrate the mass instrument with an internal calibration mode. All acquired spectra from samples were processed using TOF/TOF Explore™ Software in a default mode. The data were searched by GPS Explorer (V3.6) with the search engine MASCOT (2.1). The following parameters were used in the search: National Center for Biotechnology information non-redundant (NCBInr) database (release date, July, 2011), Sus scrofa, protein molecular mass ranged from 700 to 3,600 Da, trypsin digest with one missing cleavage, peptide tolerance of 100 ppm,MS/MS tolerance of 0.8 Da and possible oxidation of methionine. Known contaminant ions (tryptic autodigest peptides) were excluded. A total of 41,373 sequences and 16,019,616 residues in the database were actually searched. All identified proteins had a protein score greater than 59, corresponding to a statistically significant (p < 0.05) confident identification. Besides protein score, at least one ion score with p < 0.05 was recommended to increase the reliability of identifications.

### Western blot

Samples of TGEV-infected and mock-infected ST cells were lysed at 48 h p.i., and the protein concentration was determined. Equivalent amounts of cell lysates (60 μg) were subjected to 12% SDS-PAGE gels and then transferred to 0.22 μm nitrocellulose membranes (Hybond-C extra, Amersham Biosciences). After blotting, the membranes were incubated with mouse monoclonal antibody (mAb) to alpha tubulin (1:1000, Abcam), mAb to prohibitin (1:500, II-14-10, Santa Cruz), and rabbit polyclonal antibody to keratin19 (1:500, Bioss) at 37°C for 60 min. After washing three times with PBST, the membranes were inoculated with horseradish peroxidase (HRP) conjugated goat anti-mouse IgG (Kirkegaard & Perry Laboratories, Inc.) or goat anti-rabbit IgG (Kirkegaard & Perry Laboratories, Inc.) at 37°C for 60 min and visualized using 3,3′,5,5′-tetramethylbenzidine-stabilized substrate (TMB, Promega).

### Real time RT-PCR

Total RNA was extracted from the ST cells, which were infected with TGEV for 48 h, using the EZ-10 Spin Column RNA Purification Kit (Qiagen) according to the manufacturer’s protocol. cDNA synthesis was performed with 1 μg of total cellular RNA using a RevertAid™ first strand cDNA synthesis kit (Fermentas), according to manufacturer’s protocol. The specific primers for amplifying various target genes for differentially expressed proteins were designed according to the available gene sequences deposited in GenBank using Lasergene sequence analysis software (DNAStar, Inc., Madison, WI, USA) (Table 2). Real time RT-PCR was performed using a LightCycler 480 II (Roche) in a total volume of 20 μL containing 10 ng of cDNA template, 1 × SYBR® Premix Ex Taq™ II (Perfect Real Time, TaKaRa), and a 0.4 μM concentration of each primer. After initial denaturation at 95°C for 2 min, the amplification was performed for 40 cycles, each consisting of denaturation at 95°C for 5 s and primer annealing at 55°C for 15 s. Melting curves were obtained, and quantitative analysis of the data was performed in a relative quantification (2^-ΔΔCT^) study model. Parallel mock-infected ST cells were used as control (relative expression = 1) and GAPDH as an internal reference gene.

**Table 2:** Primers used for real time RT-PCR

**Gene symbol**	**Forward primers (5′-3′)**	**Reverse primers (5′-3′)**	**Length (bp)**	**Gene accession no.**
GAPDH	GGTGAAGGTCGGAGTGAACG	CGTGGGTGGAATCATACTGG	152	NM_001206359
ANXA8	AACCTCCACAGCTACTTTGCC	CATCTTGTTGAACTGACCCTTGA	138	NM_001243599
KRT19	AGCGGCAGAATCAGGAGTAC	AGAGGACCTTGGAGGCAGAC	132	NM_002276
LDHB	GGAAGATAAACTCAAGGGAGAAATG	CTGCCGTCACCACCACAAT	128	NM_001113287
PP2A	GTGGAGAAGTTTGGGAAGGAGT	AGCATGTGCTTGGTGGTGAT	158	NM_214024
TPMT	CTTCGTCGCCGTTAATCCAG	TCATAAGCCAACACGCACAAG	99	NM_001243675
UQCRC1	GAAGGAAATTGACCAGGAGG	GGGGCAGTAATAACCACC	169	XM_003127002

## Abbreviations

2D DIGE: Two-dimensional difference gel electrophoresis; ACN: Acetonitrile; CHAPS: 3-[(3-cholamidopropyl) dimethyl-ammonio]-1-propanesulfonate; DTT: Dithiothreitol; GAPDH: Glyceraldehyde-3-phosphate dehydrogenase; TGEV: Transmissible gastroenteritis coronavirus; IEF: Isoelectric focusing; IPG: Immobilized pH gradient; MALDI-TOF-TOF/MS: Matrix-assisted laser desorption/ionization time-of-flight tandem mass spectrometry; RT-PCR: Reverse transcriptase-polymerase chain reaction; PMF: Peptide mass fingerprinting; SDS-PAGE: Sodium dodecyl sulfate polyacrylamide gel electrophoresis.

## Competing interests

The authors declare that they have no competing interests.

## Authors’ contributions

LF designed the study. LF and XZ were responsible for data analysis and interpretation and drafted the manuscript; HS, JC, and DS carried out the 2D DIGE gel analysis and excised the protein spots; HL and ZW were responsible for mass spectrometry analysis and manuscript editing. All authors read and approved the final manuscript.

## Supplementary Material

Additional file 1: Figure S1 MS and MS/MS spectra of the identiifed protein spots. Click here for file

Additional file 2: Table S1 Cell proteins identified from the differential 2D DIGE analysis after TGEV infection. Click here for file
