# Supplementary material for: Identification of cellular proteome using two-dimensional difference gel electrophoresis in ST cells infected with transmissible gastroenteritis coronavirus
Source: Proteome Sci. 2013 Jul 16;11:31. doi: 10.1186/1477-5956-11-31 (PMC3734006; doi:10.1186/1477-5956-11-31)
Supplement: Additional file 2: Table S1 — Cell proteins identified from the differential 2D DIGE analysis after TGEV infection. [file 1477-5956-11-31-S2.doc]

Supplemental Table 1. Cell proteins identified from the differential 2D DIGE analysis after TGEV infection

| Spot number | Protein name | Accession number | Mass (Da) | pI | Coverage (%) | Protein score | Peptide matched | Peptide Identified (ion score) |
| --- | --- | --- | --- | --- | --- | --- | --- | --- |
| 1154 | beta actin | [gi|45269029](http://192.168.30.63/mascot/cgi/master_results.pl?file=../data/20120416/F005550.dat&REPTYPE=protein#Hit1) | 44763 | 5.55 | 36 | 285 | 3 | R.GYSFTTTAER.E (58)  K.SYELPDGQVITIGNER.F (78)  K.QEYDESGPSIVHR.K (96) |
| 1308 | beta actin | [gi|45269029](http://192.168.30.63/mascot/cgi/master_results.pl?file=../data/20120416/F005550.dat&REPTYPE=protein#Hit1) | 44763 | 5.55 | 16 | 407 | 3 | K.IWHHTFYNELR.V (55)  K.SYELPDGQVITIGNER.F (128)  K.DLYANTVLSGGTTMYPGIADR.M (180) |
| 1312 | beta actin | [gi|45269029](http://192.168.30.63/mascot/cgi/master_results.pl?file=../data/20120416/F005550.dat&REPTYPE=protein#Hit1) | 44763 | 5.55 | 14 | 129 | 3 | R.GYSFTTTAER.E (1)  K.SYELPDGQVITIGNER.F (106)  K.DLYANTVLSGGTTMYPGIADR.M (4) |
| 1368 | beta actin | [gi|45269029](http://192.168.30.63/mascot/cgi/master_results.pl?file=../data/20120416/F005549.dat&REPTYPE=protein#Hit1) | 44763 | 5.55 | 16 | 82 | 1 | K.SYELPDGQVITIGNER.F (62) |
| 1383 | beta actin | [gi|45269029](http://192.168.30.63/mascot/cgi/master_results.pl?file=../data/20120416/F005550.dat&REPTYPE=protein#Hit1) | 44763 | 5.55 | 34 | 537 | 5 | K.IWHHTFYNELR.V (58)  R.VAPEEHPVLLTEAPLNPK.A (46)  R.GYSFTTTAER.E (117)  K.SYELPDGQVITIGNER.F (93)  K.DLYANTVLSGGTTMYPGIADR.M (148) |
| 1647 | beta actin | [gi|150438831](http://192.168.30.63/mascot/cgi/master_results.pl?file=../data/20120416/F005565.dat&REPTYPE=protein#Hit1) | 44763 | 5.55 | 21 | 242 | 3 | K.IWHHTFYNELR.V (73)  K.SYELPDGQVITIGNER.F (107)  K.DLYANTVLSGGTTMYPGIADR.M (24) |
| 1675 | alpha-tropomyosin | [gi|158931149](http://192.168.30.63/mascot/cgi/master_results.pl?file=../data/20120416/F005559.dat&REPTYPE=protein#Hit1) | 32687 | 4.71 | 46 | 544 | 5 | R.RIQLVEEELDR.A (66)  R.IQLVEEELDR.A (89)  R.IQLVEEELDRAQER.L (91)  R.KLVIIESDLER.A (75)  K.LVIIESDLER.A (64) |
| 1864 | beta actin | [gi|476332](http://192.168.30.63/mascot/cgi/master_results.pl?file=../data/20120416/F005647.dat&REPTYPE=protein#Hit1) | 26102 | 5.55 | 35 | 177 | 3 | R.GYSFTTTAER.E (23)  K.SYELPDGQVITIGNER.F (112)  K.DLYANTVLSGGTTMYPGIADR.M (2) |
| 911 | alpha Tubulin | [gi|116256086](http://192.168.30.63/mascot/cgi/master_results.pl?file=../data/20120416/F005564.dat&REPTYPE=protein#Hit1) | 50120 | 4.94 | 28 | 340 | 4 | K.EIIDLVLDR.I (60)  R.QLFHPEQLITGK.E (47)  R.LISQIVSSITASLR.F (66)  R.AVFVDLEPTVIDEVR.T (117) |
| 964 | beta Tubulin | [gi|75045190](http://192.168.30.63/mascot/cgi/master_results.pl?file=../data/20120416/F005577.dat&REPTYPE=protein#Hit1) | 49639 | 4.78 | 31 | 448 | 7 | R.AILVDLEPGTMDSVR.S (57)  R.FPGQLNADLR.K (76)  K.LAVNMVPFPR.L (54)  K.LAVNMVPFPR.L (6)  R.YLTVAAVFR.G (50)  R.ISEQFTAMFR.R (21)  R.ISEQFTAMFR.R (119) |
| 978 | alpha Tubulin | [gi|116256086](http://192.168.30.63/mascot/cgi/master_results.pl?file=../data/20120416/F005574.dat&REPTYPE=protein#Hit1) | 50120 | 4.94 | 32 | 338 | 3 | K.EIIDLVLDR.I ()  R.QLFHPEQLITGK.E ()  R.AVFVDLEPTVIDEVR.T |
| 1078 | vimentin | [gi|335296459](http://192.168.30.63/mascot/cgi/master_results.pl?file=../data/20120416/F005557.dat&REPTYPE=protein#Hit1) | 53635 | 5.06 | 64 | 1020 | 6 | R.SLYTSSPGGVYATR.S (78)  R.TNEKVELQELNDR.F (91)  K.LQEETLQREEAESTLQSFR.Q (100)  K.FADLSEAANR.N (103)  R.EMEENFAVEAANYQDTIGR.L (138)  R.ISLPLPNFSSLNLR.E (133) |
| 1266 | vimentin | [gi|335296459](http://192.168.30.63/mascot/cgi/master_results.pl?file=../data/20120416/F005557.dat&REPTYPE=protein#Hit1) | 53635 | 5.06 | 60 | 888 | 6 | R.LGDLYEEEMR.E (64)  K.LQEETLQREEAESTLQSFR.Q (59)  R.EEAESTLQSFR.Q (92)  K.FADLSEAANR.N (86)  R.EMEENFAVEAANYQDTIGR.L (185)  R.ISLPLPNFSSLNLR.E (87) |
| 1892 | vimentin | [gi|335296459](http://192.168.30.63/mascot/cgi/master_results.pl?file=../data/20120416/F005557.dat&REPTYPE=protein#Hit1) | 53635 | 5.06 | 35 | 389 | 2 | R.EMEENFAVEAANYQDTIGR.L (99)  R.ISLPLPNFSSLNLR.E (176) |
| 1947 | vimentin | [gi|335296459](http://192.168.30.63/mascot/cgi/master_results.pl?file=../data/20120416/F005557.dat&REPTYPE=protein#Hit1) | 53635 | 5.05 | 35 | 582 | 4 | K.FADLSEAANR.N (74)  R.EMEENFAVEAANYQDTIGR.L (82)  R.LQDEIQNMKEEMAR.H (107)  R.ISLPLPNFSSLNLR.E (181) |
| 1968 | vimentin | [gi|21431723](http://192.168.30.63/mascot/cgi/master_results.pl?file=../data/20120416/F005683.dat&REPTYPE=protein#Hit1) | 30971 | 4.67 | 25 | 366 | 4 | R.EMEENFAVEAANYQDTIGR.L (69)  K.MALDIEIATYR.K (10)  K.MALDIEIATYR.K (92)  R.ISLPLPNFSSLNLR.E (168) |
| 1970 | vimentin | [gi|335296459](http://192.168.30.63/mascot/cgi/master_results.pl?file=../data/20120416/F005557.dat&REPTYPE=protein#Hit1) | 53635 | 5.06 | 35 | 603 | 4 | K.FADLSEAANR.N (93)  R.EMEENFAVEAANYQDTIGR.L (96)  R.LQDEIQNMKEEMAR.H (109)  R.ISLPLPNFSSLNLR.E (168) |
| 1907 | keratin 19 | [gi|311267276](http://192.168.30.63/mascot/cgi/master_results.pl?file=../data/20120416/F005561.dat&REPTYPE=protein#Hit1) | 44186 | 5.05 | 47 | 682 | 8 | R.QSSATSSFGGLGGGSVR.F (43)  K.LTMQNLNDR.L (51)  K.LTMQNLNDR.L (56)  K.IRDWYQK.Q (52)  K.IVLQIDNAR.L (25)  R.TKFETEQALR.L (81)  R.LSVEADINGLRR.V (53)  R.VLDELTLAR.T (145) |
| 2090 | vimentin | [gi|21431723](http://192.168.30.63/mascot/cgi/master_results.pl?file=../data/20120416/F005566.dat&REPTYPE=protein#Hit1) | 30971 | 6.47 | 9 | 120 | 2 | K.MALDIEIATYR.K (26)  R.ISLPLPNFSSLNLR.E (87) |
| 2280 | vimentin | [gi|335296459](http://192.168.30.63/mascot/cgi/master_results.pl?file=../data/20120416/F005557.dat&REPTYPE=protein#Hit1) | 53635 | 5.06 | 34 | 504 | 6 | R.TNEKVELQELNDR.F (54)  K.VELQELNDR.F (61)  R.LGDLYEEEMR.E (79)  R.LGDLYEEEMR.E (47)  K.LQEETLQR.E (96)  R.EEAESTLQSFR.Q (103) |
| 1606 | heterogeneous nuclear ribonucleoprotein U | [gi|335296158](http://192.168.30.63/mascot/cgi/master_results.pl?file=../data/20120416/F005560.dat&REPTYPE=protein#Hit1) | 104848 | 5.85 | 7 | 277 | 3 | K.DIDIHEVR.I (59)  K.NGQDLGIAFK.I (72)  K.EKPYFPIPEEYTFIQNVPLEDR.V (135) |
| 1803 | thiopurine S-methyltransferase | [gi|311259781](http://192.168.30.63/mascot/cgi/master_results.pl?file=../data/20120416/F005563.dat&REPTYPE=protein#Hit1) | 28456 | 5.46 | 41 | 497 | 5 | R.NISFHQER.G (53)  R.GHSVVGVEISELGIR.E (36)  R.GAFVAVNPGDR.K (81)  K.HAGPPFYVPDAEIER.L (136)  K.VDVFEER.H (107) |
| 2023 | thiopurine S-methyltransferase | [gi|311259781](http://192.168.30.63/mascot/cgi/master_results.pl?file=../data/20120416/F005584.dat&REPTYPE=protein#Hit1) | 28456 | 5.46 | 54 | 476 | 4 | K.NRMLTLEEWQEK.W (61)  R.NISFHQER.G (74)  R.GHSVVGVEISELGIR.E (121)  K.HAGPPFYVPDAEIER.L (111) |
| 716 | protein phosphatase 2A | [gi|510469](http://192.168.30.63/mascot/cgi/protein_view.pl?file=../data/20120416/F005562.dat&hit=3) | 65281 | 5.00 | 30 | 239 | 2 | K.LSTIALALGVER.T (79)  R.LAGGDWFTSR.T (64) |
| 1116 | eukaryotic initiation factor 4A-I | [gi|154147660](http://192.168.30.63/mascot/cgi/master_results.pl?file=../data/20120416/F005585.dat&REPTYPE=protein#Hit1) | 46125 | 5.32 | 44 | 586 | 7 | R.GIYAYGFEKPSAIQQR.A (33)  K.LQMEAPHIIVGTPGR.V (5)  R.VFDMLNR.R (56)  R.VFDMLNR.R (2)  K.MFVLDEADEMLSR.G (90)  R.QFYINVER.E (110)  R.GIDVQQVSLVINYDLPTNR.E (142) |
| 1158 | eukaryotic initiation factor 4A-I | [gi|154147660](http://192.168.30.63/mascot/cgi/protein_view.pl?file=../data/20120416/F005569.dat&hit=1) | 46125 | 5.32 | 40 | 531 | 5 | R.GIYAYGFEKPSAIQQR.A (48)  K.LQMEAPHIIVGTPGR.V (56)  R.VFDMLNR.R (84)  R.QFYINVER.E (96)  R.GIDVQQVSLVINYDLPTNR.E (120) |
| 1509 | acidic ribosomal protein P0 | [gi|182705234](http://192.168.30.63/mascot/cgi/master_results.pl?file=../data/20120416/F005646.dat&REPTYPE=protein#Hit1) | 34337 | 5.71 | 50 | 483 | 4 | K.IIQLLDDYPK.C (47)  R.GNVGFVFTKEDLTEIR.D (50)  K.TSFFQALGITTK.I (121)  K.AFLADPSAFVAAAPVAAATTAAPAAAAAAPAK.V (148) |
| 1602 | annexin A8 | [gi|194042330](http://192.168.30.63/mascot/cgi/master_results.pl?file=../data/20120416/F005570.dat&REPTYPE=protein#Hit1) | 36705 | 5.20 | 63 | 752 | 6 | K.GSPHFNPDPDAETLYK.A (72)  K.GIGTNEQAIIDVLTKR.S (72)  R.LIIALMYPPYK.Y (31)  K.EGVIIEILASR.T (85)  K.AYEEDYGSSLEEDIQADTSGYLER.I (96)  R.NLHSYFAER.L (213) |
| 1814 | annexin A4 | [gi|4033507](http://192.168.30.63/mascot/cgi/master_results.pl?file=../data/20120416/F005578.dat&REPTYPE=protein#Hit1) | 35806 | 5.71 | 49 | 510 | 5 | K.AASGFNAAEDAQTLR.K (47)  K.GLGTDEDAIISVLAYR.S (34)  R.INQTYQLQYGR.S (74)  R.SDTSFMFQR.V (129)  R.SDTSFMFQR.V (128) |
| 906 | cytochrome b-c1 complex subunit 1 | [gi|335299041](http://192.168.30.63/mascot/cgi/master_results.pl?file=../data/20120416/F005588.dat&REPTYPE=protein#Hit1) | 52665 | 5.76 | 38 | 342 | 4 | R.DVILQELQENDSSMR.D (28)  R.RIPLAEWESR.I (3)  R.SGMFWLR.F (63)  R.SGMFWLR.F (104) |
| 1094 | prohibitin | [gi|335308255](http://192.168.30.63/mascot/cgi/master_results.pl?file=../data/20120416/F005649.dat&REPTYPE=protein#Hit1) | 28877 | 5.74 | 72 | 688 | 6 | K.FGLALAVAGGVVNSALYNVDAGHR.A (70)  K.DLQNVNITLR.I (77)  R.ILFRPVASQLPR.I (32)  R.IFTSIGEDYDER.V (88)  R.FDAGELITQR.E (126)  R.KLEAAEDIAYQLSR.S (127) |
| 1871 | 14-3-3 protein eta | [gi|194043292](http://192.168.30.63/mascot/cgi/master_results.pl?file=../data/20120416/F005576.dat&REPTYPE=protein#Hit1) | 28194 | 4.81 | 30 | 282 | 4 | K.AVTELNEPLSNEDR.N (61)  K.AVTELNEPLSNEDRNLLSVAYK.N (8)  K.EHMQPTHPIR.L (84)  K.DSTLIMQLLR.D (72) |
| 1640 | L-lactate dehydrogenase B chain | [gi|164518958](http://192.168.30.63/mascot/cgi/protein_view.pl?file=../data/20120416/F005567.dat&hit=3) | 36424 | 6.73 | 22 | 126 | 2 | K.IVVVTAGVR.Q (54)  K.FIIPQIVK.Y (33) |
